# Exploring the Interaction of Curaxin CBL0137 with G-Quadruplex DNA Oligomers

**DOI:** 10.3390/ijms22126476

**Published:** 2021-06-17

**Authors:** Sabrina Dallavalle, Luce M. Mattio, Roberto Artali, Loana Musso, Anna Aviñó, Carme Fàbrega, Ramon Eritja, Raimundo Gargallo, Stefania Mazzini

**Affiliations:** 1 Department of Food, Environmental and Nutritional Sciences (DEFENS), University of Milan (Università degli Studi di Milano), 20133 Milan, Italy; sabrina.dallavalle@unimi.it (S.D.); luce.mattio@unimi.it (L.M.M.); loana.musso@unimi.it (L.M.); 2 National Institute of Fundamental Studies, Kandy 20000, Sri Lanka; 3 Scientia Advice di Roberto Artali, 20832 Desio, Italy; roberto.artali@scientia-advice.com; 4 Institute for Advanced Chemistry of Catalonia (IQAC), CSIC, Networking Center on Bioengineering, Biomaterials and Nanomedicine (CIBER-BBN), 08034 Barcelona, Spain; aaagma@cid.csic.es (A.A.); carme.fabrega@iqac.csic.es (C.F.); recgma@cid.csic.es (R.E.); 5 Department of Chemical Engineering and Analytical Chemistry, University of Barcelona, 08028 Barcelona, Spain; raimon_gargallo@ub.edu

**Keywords:** curaxin, NMR spectroscopy, circular dichroism, G-quadruplex, molecular modeling, DNA interactions, *c*-*myc*

## Abstract

Curaxins and especially the second-generation derivative curaxin CBL0137 have important antitumor activities in multiple cancers such as glioblastoma, melanoma and others. Although most of the authors suggest that their mechanism of action comes from the activation of p53 and inactivation of NF-kB by targeting FACT, there is evidence supporting the involvement of DNA binding in their antitumor activity. In this work, the DNA binding properties of curaxin CBL0137 with model quadruplex DNA oligomers were studied by ^1^H NMR, CD, fluorescence and molecular modeling. We provided molecular details of the interaction of curaxin with two G-quadruplex structures, the single repeat of human telomere d(TTAGGGT)_4_ and the *c*-*myc* promoter Pu22 sequence. We also performed ^1^H and ^31^P NMR experiments were also performed in order to investigate the interaction with duplex DNA models. Our data support the hypothesis that the interaction of curaxin with G-quadruplex may provide a novel insight into the DNA-binding properties of CBL0137, and it will be helpful for the design of novel selective DNA-targeting curaxin analogues.

## 1. Introduction

**Figure 1:**
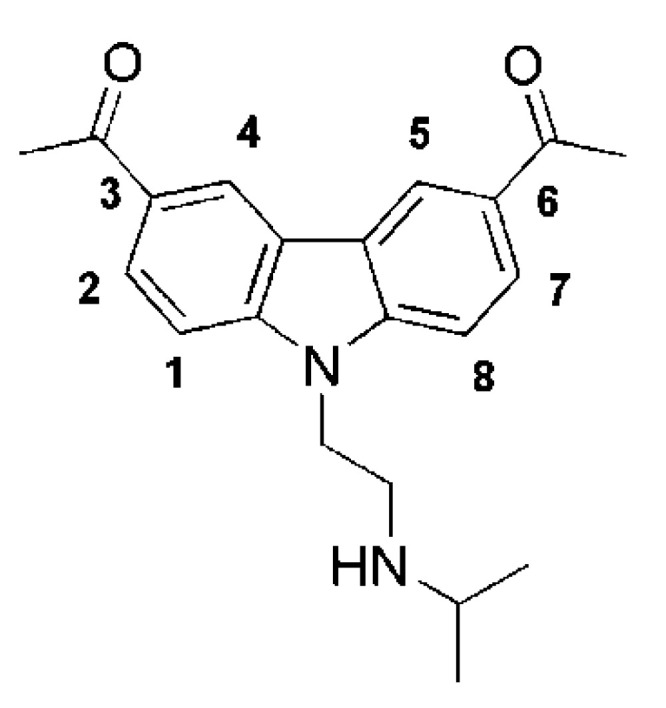
Structure of curaxin CBL0137.

Curaxins are a small group of substances endowed with anticancer activity [1]. They were identified in a search for non-genotoxic antiproliferative compounds, simultaneously acting on two tumor targets. The first hits found were quinacrine and other antimalarials, but SAR studies led rapidly to more active compounds, represented by curaxins, a class of substituted carbazoles with electron-withdrawing groups at positions 3 and 6, and an aminoalkyl chain at the nitrogen 9. Curaxins are able to activate the tumor suppressing protein p53 and to suppress the anti-apoptotic nuclear factor NF-κB [2,3]. Importantly, curaxins were found to be more toxic to tumor than to normal cells. Among curaxins, CBL0137 (Figure 1), appeared the most suitable for further development, on the basis of its metabolic stability, solubility and activity in vivo. CBL0137 suppressed tumor growth in xenograft models of colon (DLD-1), renal cell carcinoma (Caki-1) [3], medulloblastoma [4], small-cell lung cancer [5,6], melanoma (Mel-7) and transplanted surgical samples from patients with pancreatic ductal adenocarcinoma [7].

CBL0137 was also found to be active in mixed-lineage leukemia [8] and against cancer stem cells and to potentiate efficacy of gemcitabine in pancreatic cancer [9].

At present, CBL0137 is under phase I clinical trials in patients with hematological malignancies and solid tumors [10].

The cytotoxicity of curaxins, which are associated with the absence of DNA damage, stimulated studies directed to unveil their mechanism of action. Eukaryotic DNA is packed into chromatin, which is a highly ordered complex of DNA and histone proteins. Its basic unit, the nucleosome, consists of an octamer of histones, known as the histone or nucleosome core, which is bound to a 147 base pairs of DNA fragment.

Recent studies suggested that CBL0137 interacts with DNA by modifying the shape of the DNA helix, thus increasing the interbase-pair distance. As a consequence, DNA unwinding and detachment from the histone octamer occur, eventually leading to nucleosome disassembly both in vitro and in cells. The destabilization of the nucleosomes induces the intervention of a histone chaperone, FACT (Facilitates Chromatin Transcription), which binds tightly to chromatin (c-trapping) [2,11,12]. This results in activating phosphorylation of p53 by FACT-associated CK2 and reducing NF-kB–dependent transcription, because of depletion of soluble FACT. Recently, it was reported that such effects are associated with the induction of the interferon response to epigenetic derepression of the cellular “repeatome” [13].

Kantidze et al. [14] found that CBL0137 alters DNA topology leading to the inability of the transcriptional repressor CTCF to bind efficiently to its cognate DNA sites. This effect on CTCF binding results in partial disruption of chromatin loops and in large-scale perturbations in the 3D genome organization.

More recently, Lu et al. [15] tried to clarify how curaxins alter the genomic DNA structure and affect the DNA binding property of key proteins, such as CTCF and FACT. They found that CBL0137 strongly and persistently binds to dsDNA, inducing a huge barrier for DNA unzipping during replication and transcription, thus causing the distinct binding response of CTCF and FACT on DNA.

Further investigations revealed that several pathways, such as inhibition of the self-renewal of cancer stem cells/tumor-initiating cells through NOTCH1 activation and downregulation of heat shock factor 1 (HSF1), thereby increasing tumor cell apoptosis, are involved in the process [5,14,16]. Moreover, CBL037 highly suppresses the expression of c-MYC family genes. Sergeev et al. [17] reported that this curaxin significantly inhibits in vitro DNA methylation by eukaryotic DNA methyltransferase Dnmt3a at low micromolar concentrations. These effects are attributed to the intercalation of CBL0137 into DNA [3,11], although there is no direct evidence for this type of interaction. The change of topology of DNA by binding with CBL0137 was deduced from CD experiments [11], whereas molecular modeling studies showed a possible protruding of the side chains of the carbazole nucleus into the major groove of DNA, with the carbazole *N*-side chain filling the minor groove [11].

The above reported effects, including inhibition of c-MYC expression, DNA methyltransferase inhibition, and chromatin remodelling, would be consistent with DNA G-quadruplex binding [18,19].

G-quadruplexes are non-canonical nucleic acids secondary structures that may form in G-rich sequences under physiological conditions. Their structural building block is the G-quartet, a planar array of four guanines paired through Hoogsteen bonds. G-quadruplexes play a role in several key cellular processes, including gene transcription, chromatin epigenetics and DNA recombination. G-quadruplex DNA is found in key regulatory regions of the cell such as promoters of proto-oncogenes (*c-myc*, *bcl-2* and *c-Kit*) [20]. Stabilization of the folded G-quadruplexes due to ligand interactions is proposed to inhibit the binding of transcription factors, leading to downstream silencing of oncogene expression [21,22,23]. Notably, also human telomeric sequences are able to form G-quadruplex structures that are not recognized by telomerase, an enzyme involved in telomere elongation [24].

The planar structure with an aminoalkyl side chain of CBL0137, similar to other non-DNA-damaging G-quadruplex ligands [23], supports the hypothesis that G-quadruplex interaction can have a role in the curaxin activity; however, so far, no evidence has been given for such an interaction.

To confirm our hypothesis, we undertook an investigation of the binding of CBL0137 to G-quadruplex DNA structures of telomeres and promoter oncogenes by exploiting fluorescence spectroscopy, CD, NMR and molecular modelling. We used as models the single repeat sequence of human telomere, d(TTAGGGT)_4_, and the G to T mutated Pu22 sequence (Pu22T14T23) of the *c-myc* oncogene, which is overexpressed in a wide range of human tumors.

To further investigate the possible intercalation mode of curaxin into the double helix DNA, as previously reported by some authors (References [3,11]), the study was extended to the self-complementary double helix oligomers d(CGTACG)_2_ and d(AAGAATTCTT)_2_ as models for CG and AT-rich sequences, respectively.

Our work provides a novel insight into the DNA-binding properties of CBL0137 that may be relevant in the important anticancer activity of curaxines.

## 2. Results and Discussion

To increase the solubility of CBL0137 into the aqueous medium, its hydrochloric salt **1** was prepared by treatment of the free amine with HCl in dioxane.

### 2.1. Interaction of Curaxin with Telomere d(TTAGGGT)_4_ Quadruplex

**Figure 2:**
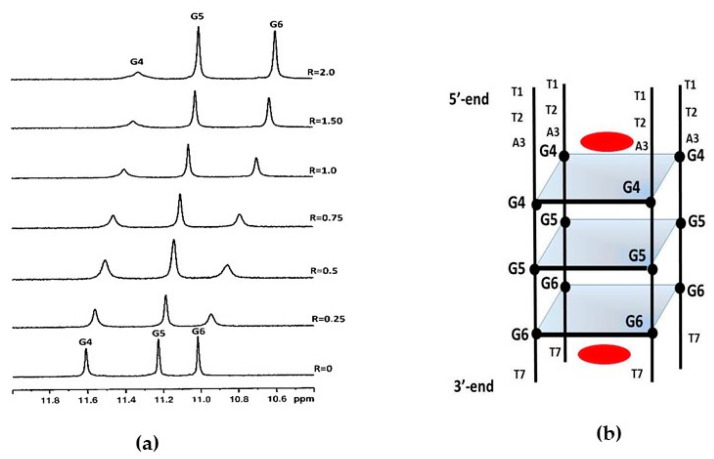
(**a**) Imino proton region of the 1D NMR titration spectra of d(TTAGGGT)_4_ with curaxin at 25 °C in H_2_O/D_2_O (9:1), 25 mM KH_2_PO_4_, 150 mM KCl and 1 mM EDTA, pH 6.7, at different R = [drug]/[DNA] ratios; (**b**) schematic representation of d(TTAGGGT)_4_/curaxin (in red) complex.

The imino proton region of the ^1^H NMR spectrum of d(TTAGGGT)_4_ showed three signals between 10 and 12 ppm that are indicative of the formation of a single G-quadruplex species with three G-quartet planes. As curaxin was added to the d(TTAGGGT)_4_ solution, the NH imino protons moved upfield and G4 and G6 signals became broad even at low ratio R = [curaxin]/[DNA] = 0.25/0.75. At R = 2.0 the imino proton of G4 remained very broad, while the G6 became sharp again. This behavior can be explained by the binding of curaxin to G4 and G6 tetrads, with an intermediate exchange between free and bound state at the level of G4. The greatest variation of chemical shift is observed for G6 signal (Δδ = −0.55 ppm) (Figure 2).

**Figure 3:**
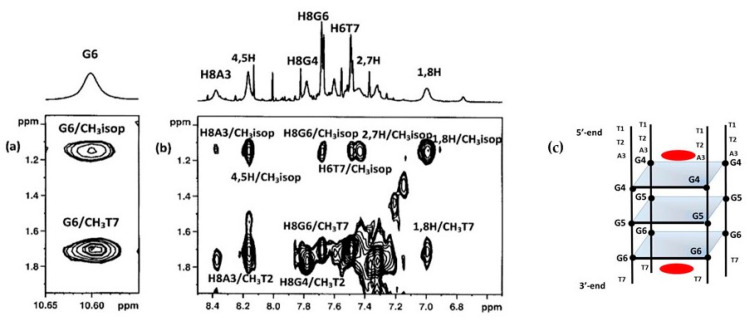
Selected region of the 2D NOESY spectrum of d(TTAGGGT)_4_/curaxin complex. (**a**) G6 imino proton displays intermolecular NOEs between CH_3_T7 and CH_3_(isopropyl) of curaxin; (**b**) A3, G6 and T7 aromatic protons and methyl groups of T2 and T7 of T2AG3T display intermolecular NOEs with CH_3_ (isopropyl) and aromatic protons of curaxin; (**c**) schematic representation of d(TTAGGGT)_4_/curaxin (in red) complex.

In order to better define the geometry of the complex, a series of 2D NMR experiments were performed. NOESY and TOCSY experiments allowed us to identify the curaxin (Table S1) and d(TTAGGGT)_4_ protons in the complex (Table S2). Despite of the overlapping of some of curaxin and oligonucleotide signals, several intermolecular NOE interactions were detected. The contacts involved both aromatic and side chain protons of the curaxin with aromatic and ribose protons of d(TTAGGGT)_4_ at A3G4 and G6T7 sites (Table 1 and Figure 3).

**Figure 4:**
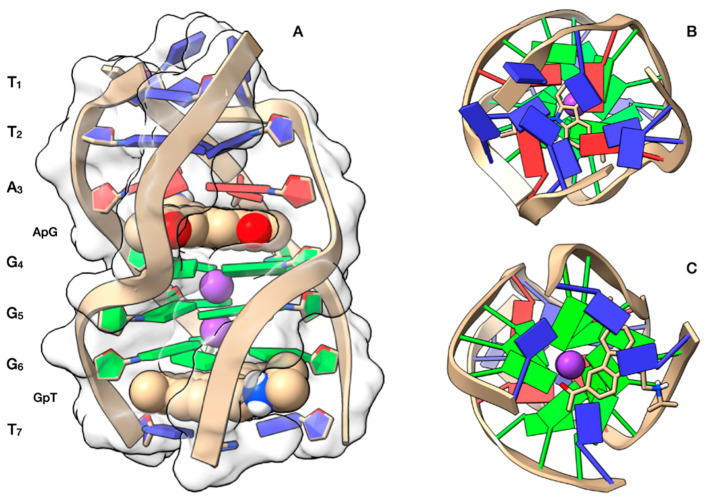
Graphical representations of the curaxin-d(TTAGGGT)_4_ complexes at the ApG and GpT intercalation sites, obtained by Molecular Docking and optimized by Molecular Dynamics (MD). (**A**) Side view of the ghostly white solvent accessible surface (SAS) of the d(TTAGGGT)_4_ quadruplex. The nucleotides are represented in stick and filled rings: adenine in red, guanine in green and thymine in blue. The ligand is represented as van der Waals (vdW) spheres and colored following the CPK code. The optimized conformations of the ligand are represented in (**B**) for the complex at ApG and in (**C**) for the complex at GpT. Potassium ions are represented by their vdW spheres (K^+^ in purple), while the ligand is depicted in stick and colored following the CPK code. The nucleotides are represented as filled plates: adenine in red, guanine in green and thymine in blue.

The NMR studies were complemented by a molecular docking simulation, followed by molecular dynamics (MD) optimization. Curaxin was docked at both A3G4 and G6T7 sites (Figure 4A). At the A3G4 binding site the ligand adopts a quite centre-symmetrical location, allowing the formation of π-π stacking interactions with all the bases of the upper A3 and lower G4 tetrads. In this site the complex is stabilized by a hydrogen bond between the charged quaternary nitrogen group of curaxin and N_7_A3, at a distance of 2.44 Å. One of the two benzene rings of curaxin lies above the K^+^ ion, resulting in a strong cation-π interaction (4.90 Å). The interaction pattern is completed by two other cation-π interactions formed between the quaternary nitrogen of the ligand and the aromatic component of the A3 and G4 bases (Figure 4B).

Otherwise, at the G6T7 site the ligand does not adopt a center-symmetrical stacking interaction but it is rather shifted towards one half of the G6 tetrad (Figure 4C). In this orientation, the ligand gives π-π stacking interactions with G6 and T7 and is stabilized by an attractive charge interaction between OP_2_G6 and the charged quaternary nitrogen group of the ligand. The quaternary nitrogen is also involved in a cation-π interaction with the aromatic component of T7.

The best docked conformations of the complexes at the ApG and GpT intercalation sites are in good agreement with the reported NOE contacts (Table 1).

### 2.2. Interaction of Curaxin with Pu22T14T23 G-Quadruplex

**Figure 5:**
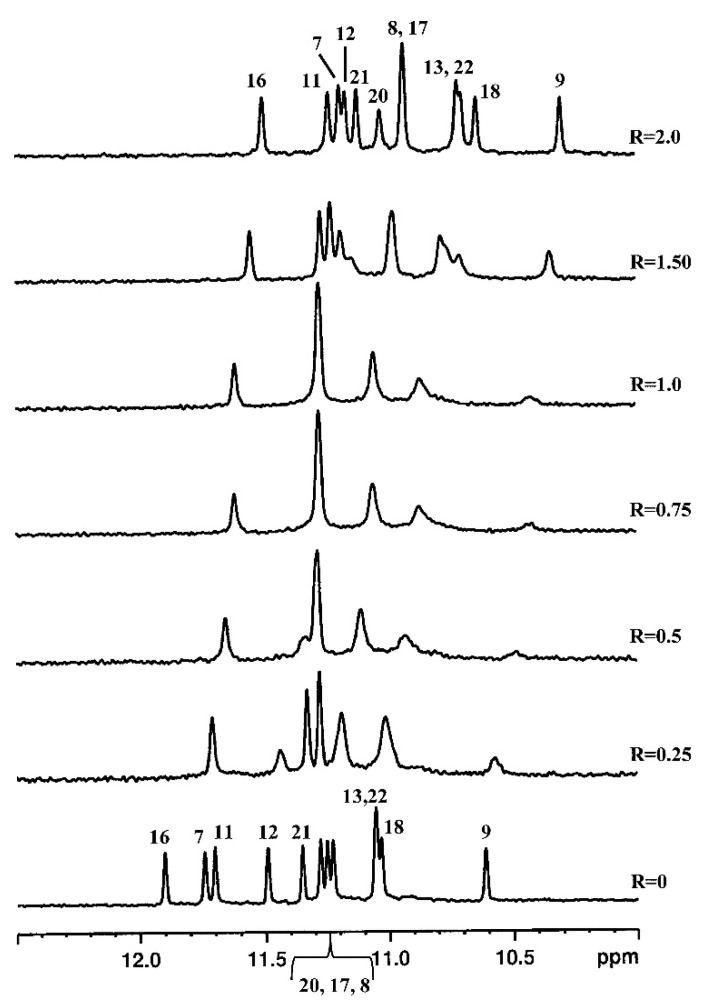
Imino proton region of the 1D NMR titration spectra of Pu22T14T23 with curaxin at 25 °C in H_2_O/D_2_O (9:1), 25 mM KH_2_PO_4_ and 70 mM KCl at pH 6.9, at different R = [drug]/[DNA] ratios.

An important and generalized line broadening of the guanine NH imino protons was observed upon the titration of Pu22T14T23 with curaxin, even at low ratio R = 0.25/1.0. This can be due to a strong interaction of the curaxin with the nucleotide, producing an intermediate exchange process between free and bound state on the NMR timescale. In particular, the NH signals belonging to the tetrad G9-G13-G18-G22 disappeared almost completely. At a ratio R > 1.0, the NH signals sharpened and for R = 2.0 only one set of resonance was present (Figure 5). By increasing the R value until a ratio of 4, the spectrum did not change significantly. All the signals moved upfield, but the most relevant chemical shift variation was observed for G7, G11 and G16 residues at 5′-end, and for G22, G18 and G13 at 3′-end. These findings indicate that a single conformation of the complex occurs in solution and suggest that the binding sites are at the level of the external tetrads.

The proton assignment and the inter-residue NOE connectivities characterizing the three tetrads in the complex are described in the Experimental section and the values are reported in Tables S3 and S4.

**Figure 6:**
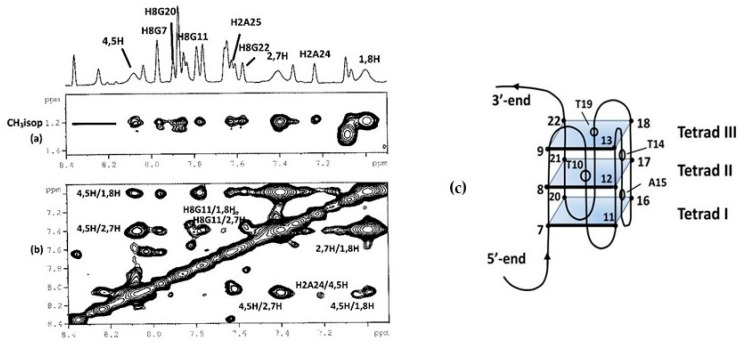
Selected region of the 2D NOESY spectrum of Pu22T14T23/curaxin complex. (**a**) Some aromatic protons of Pu22T14T23 display intermolecular NOEs with CH_3_(isopropyl) of curaxin; (**b**) some aromatic protons of Pu22T14T23 display intermolecular NOEs with aromatic protons of curaxin and (**c**) schematic representation of Pu22-T14 T23 oligomer G-quadruplex.

Many NOE interactions between curaxin and the nucleotide were revealed in the NOESY spectra (Table 2, Figure 6 and Figure S1). A large portion of the curaxin molecule, going from H1 and H2 to H7 and H8, including the side chain at N_9_, has contacts with the aromatic protons of the guanines G7, G11 and G20 at 5′-end. At the same terminal, the CH_3_CO groups of curaxin show strong interactions with the imino H1 protons of the guanines of the above tetrad.

The 3′-end terminal appears more compact. H4,5 and CH_3_CO groups present strong NOE interactions with the imino protons H1 of G18 and H1 of G22 and/or G13, respectively. In addition, both aromatic and methyl protons show significant contacts with the tail of the flanking chain A24 and A25.

These results indicate that curaxin binds the Pu22T14T23 quadruplex over the two external tetrads. The location at 3′-end appears more stable, while the interaction at 5′-end terminal seems to be characterized by a higher mobility of the ligand. This relative mobility is also suggested by the finding of additional weak NOE interactions involving the ribose H1′ proton of G7 and T4 units with H1,8 and the side chain of the ligand.

**Figure 7:**
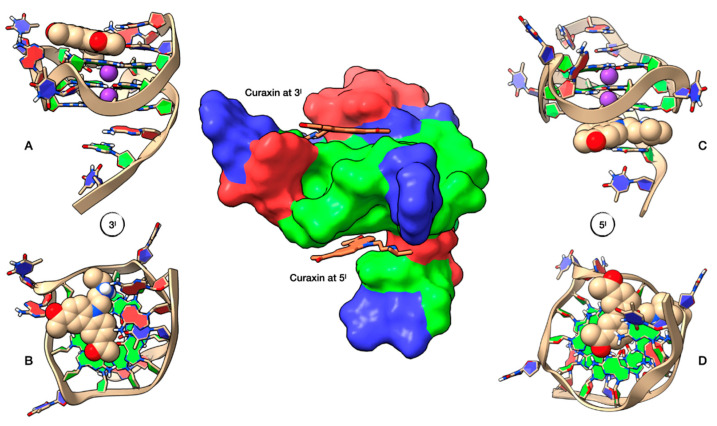
Representation of the Pu22T14T23 complexes with curaxin at 5’-end and 3’-end, obtained by Molecular Docking and optimized by Molecular Dynamics (MD). At the center of the figure Pu22T14T23, represented by its solvent accessible surface (SAS), color-coded by the underlying nucleotide (adenine in red, guanine in green and thymine in blue) and complexed with curaxin (represented in stick) at both 3’-end and 5’-end. On the left, lateral (**A**) and top (**B**) representation of the ligand conformation at the 3’-end, while on the right we can see the lateral (**C**) and bottom (**D**) representation of curaxin at the 5’-end. Ligand and potassium ions are represented by their van der Waals spheres (ligand colored in CPK, K^+^ in purple), while the nucleotide units of Pu22T14T23 are represented as filled rings: adenine in red, guanine in green and thymine in blue.

The three-dimensional models for the curaxin-Pu22T14T23 complexes were obtained by performing molecular docking experiments, followed by a Molecular Dynamics (MD) optimization of the resulting complexes (Figure 7). In both 3′-end and 5′-end positions, the curaxin molecule is arranged along the main groove of Pu22.

At 3’-end, the ligand is positioned towards the center of the tetrad and is stabilized by a π–π interaction involving G13 and one of the two benzene rings. The side chain faces A25, forming two hydrogen bonds between the quaternary nitrogen protons and N_1_A25, with distances of 2.69 Å and 2.57 Å. This explains the downfield shift of A25 H2 (Δδ + 0.21 ppm), which has lost the shielding by the aromatic system of the guanine G22. No particular interactions involving the two CH_3_CO residues are observed. (Figure 7A,B).

At 5’-end, curaxin is stabilized by an extensive network of π–π interactions involving the underlying 5’-end G-tetrad, with the tricyclic moiety located near the center of the tetrad (Figure 7C,D). The aromatic system interacts with the π systems of G5, G7, G11 and G16. The complex is held in place by two cation-π interaction. The first between the potassium ion and the pyrrole moiety (4.93 Å) and the second between the quaternary nitrogen of the side chain and the aromatic system of G7. The system is further stabilized by a hydrogen bond between one carbonyl group (CH_3_CO) and G7 H1 (3.06 Å), and by a bidentate hydrogen bond between the quaternary nitrogen of the curaxin and G5 N_7_ (2.82 Å) and A6 N1 (2.70 Å).

The best conformations of the complexes at 5’-end and 3’-end are in agreement with the reported NOE contacts (Table 2).

### 2.3. CD and Fluorescence Studies of the Complexes between Curaxin and PuT14T23 and d(TTAGGGT)_4_

The CD spectra of Pu22T14T23 showed a negative band at 245 nm and a positive band at 262 nm, which are characteristic of a parallel G-quadruplex topology. Upon heating, the G-quadruplex structure unfolded with a T_m_ equal to 89 ± 1 °C, which indicates a high thermal stability of this structure at the experimental conditions (Figure S2a). The addition of curaxin did not affect the intensity and the shape of the CD spectra of the G-quadruplex (Figure S2b). The ellipticity trace at 265 nm (Figure S2c) indicates clearly that the ligand did not compromise the stability of the structure. The determined Tm value (91 °C) suggests that curaxin produces a certain stabilization, preserving the overall G-quadruplex structure.

Considering the high stability of the tested G-quadruplex structure, CD analysis was performed also without added KCl (5 mM potassium phosphate buffer, pH 7.1), so to better investigate the compound effect. The Tm values in absence and presence of curaxine (1:3 molar ratio) were 70.0 ± 0.3 and 75.1 ± 0.4 °C, respectively (Figure S3). This confirmed that curaxine effectively stabilizes the folded G-quadruplex structure.

**Figure 8:**
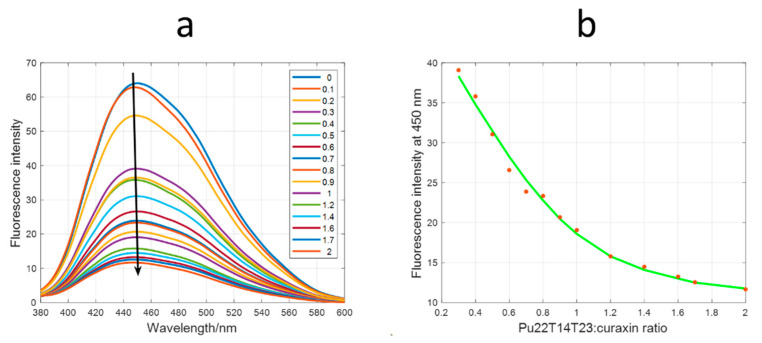
Molecular fluorescence-monitored titration of curaxin with Pu22T14T23. (**a**) Experimental spectra measured along the titration. Numbers in inset indicate the DNA:curaxin ratio; (**b**) experimental (symbols) and fitted (line) fluorescence at 450 nm. A 1:1 (DNA:curaxin) stoichiometry was used to model the data.

The fluorescence-monitored titration involved both the titration of curaxin with Pu22T14T23 and the titration of Pu22T14T23 with curaxin (Figure 8a and Figure S4a, respectively). In Figure 8a, a decreasing of the fluorescence signal intensity is observed, whereas the opposite occurs in Figure S4a, where an increasing of the fluorescence signal intensity is detected. In both cases, the estimation of the stoichiometry and the binding constants (Kb) relative to the interaction with curaxin were performed with the EQUISPEC program, which is based on the multivariate analysis of the whole spectra measured along the titration. When DNA is the titrating agent, the 1:1 complex is favored over the formation of complexes with higher stoichiometries (Figure 8b). On the contrary, complexes with a higher number of curaxin molecules occur when the DNA is titrated with the ligand [25]. From the titration curves, Kb equal to 5.2 ± 1.3 × 10^6^ M^−1^ and 7.8 ± 2.4 × 10^12^ M^−2^ were calculated for the 1:1 and 1:2 (DNA:curaxin) complexes, respectively.

Similar results were obtained when the interaction of curaxin with d(TTAGGGT)_4_ was studied.

The titrations involving curaxin and d(TTAGGGT)_4_ showed similar trends to the interaction with Pu22T14T23. Hence, the addition of DNA to curaxin produced a decrease of fluorescence, whereas the opposite effect was observed along the titration of curaxin with DNA. The data analysis was carried out in a similar way to Pu22T14T23, showing the formation of 1:1 and 1:2 (DNA:curaxin) complexes with Kb equal to 1.0 ± 1.3 × 10^6^ M^−1^ and 10.2 ± 1.0 × 10^10^ M^−2^, respectively. These values are slightly lower than in the case of those calculated for the interaction with Pu22T14T23.

The titration studies were also performed on non-G-quadruplex DNA oligonucleotides, both single and double stranded. The results of fluorescence-monitored titration of curaxine with the self-complementary sequence 5′-CGTACG-3′ showed the formation of a 1:1 complex with a binding constant of 2.0·10^6^ × M^−1^ (Figure S5). When an unfolded strand (5′-CTCTCTACTACCCTTCTGCTC-3′) was titrated, the fluorescence decrease of the ligand upon addition of DNA was clearly lower (Figure S6). This fact could reflect a weaker interaction of the ligand with the unfolded strand than with the G-quadruplex or duplex structure.

### 2.4. ^1^H and ^31^P NMR of Curaxin with Double Helix B-DNA d(CGTACG)_2_ and d(AAGAATTCTT)_2_

To further investigate the intercalation mode of curaxin into the double-helix DNA, the study was extended to models of CG and AT-rich sequences, the self-complementary double helix oligomers d(CGTACG)_2_ and d(AAGAATTCTT)_2_, respectively.

In the ^1^H NMR spectra, both B-DNA oligonucleotides displayed, in the ^1^H NMR spectra, signals from 12 to 14 ppm, assigned to the NH imino protons of the GC and AT base pairs, confirming that both oligomers adopt a double-helix conformation.

The titration of d(CGTACG)_2_ with curaxin showed, at first, the appearance in the region of 12–14 ppm, of three sets of NH imino signals (Figure S7). Increasing the R value until 3.0, the three sets of signals collapsed in one pattern of three signals assigned to a bound species. This is explained by the formation of two different complexes in slow chemical exchange, on the NMR time scale, together with the presence of the free species (Figure S7). The final situation at R = 3 shows a single complex, with up field shift of the aromatic protons of G2 and C5 units (Table S8). This suggests a binding of curaxin at the level of these residues validated by the NOE interactions between aromatic protons of curaxin with aromatic protons G2, T3 and A4 (Table S6).

The ^31^P NMR spectra of d(CGTACG)_2_ with curaxin showed a significant low field shift of all the signals. It is known that ^31^P resonance is a sensitive probe to detect changes in the phosphorus chain of the oligonucleotide [26,27]. Thus, a low field shift is an indication of intercalation processes. In our case, the significant shift found for all the signals showed that the intercalation of curaxin occurs at different sites, with an exchange among them, suggested by the broadening of the signals (Figure S8).

As concerns the interaction of curaxin with AT-rich sequence, the titration experiment led to a generalized line broadening of ^1^H and ^31^P signals (Figure S8). At R ≥ 1.0, a precipitate did not allow us to complete the experiment. The broadening of the signals should suggest some kind of interaction, but no other information can be deduced. The ^31^P spectra present insignificant shift variation, excluding an intercalation process.

## 3. Materials and Methods

### 3.1. Ligand

Curaxin CBL0137 was purchased from Biosynth Carbosynth. The corresponding hydrochloride **1** was prepared by treatment with HCl 4M in dioxane and was used for the titration experiments.

To a solution of curaxin CBL0137 (5 mg, 0.015 mmoli) in dioxane (0.4 mL), 4M HCl in dioxane (5 µL) was added, and the mixture was stirred 30 min, at RT. The solvent was removed to obtain 5.5 mg of desired compound.

### 3.2. Nuclear Magnetic Resonance

The NMR sample of G-quadruplex Pu22T14T23 d(TGAGGGTGGGTAGGGTGGGTAA) was prepared at 0.34 mM concentration in 25 mM KH_2_PO_4_, 70 mM KCl, pH 6.9, 10% D_2_O. d(TTAGGGT)_4_ was prepared at 0.45 mM concentration in G-quadruplex, in 25 mM KH_2_PO4, 150 mM KCl and 1 mM EDTA, pH 6.7, 10% D_2_O. The NMR samples of double helixes d(CGTACG)_2_ and d(AAGAATTCTT)_2_ were prepared at 0.3 mM concentration in 10 mM NaH_2_PO_4_, 100 mM NaCl, pH 7.0, 10% D_2_O. The oligonucleotide samples were heated to 85 °C for 1 min and then cooled at room temperature overnight. Curaxin was dissolved in DMSO-*d_6_* at concentration of 27 mM.

We acquired ^1^H NMR spectra at 15 and 25 °C with a Bruker AV600, 600MHz spectrometer, equipped with a TXI probe with *z*-gradient, and processed with TOPSPIN 2.1 software. We recorded ^31^P NMR spectra at 242.94 MHz with a broad-band probe and referenced at MDA (external reference). Two-dimensional NOESY spectra were acquired with mixing time between 150 and 400 ms and 2D TOCSY spectra with mixing time of 60 ms. Heteronuclear one-bond ^1^H/^13^C (HSQC) was carried out in ^1^H detection mode with broad band decoupling in the ^13^C domain.

Proton resonance assignments of the free d(TTAGGGT)_4_ [28], Pu22T14T23 [29], d(CGTACG)_2_ [30] and d(AAGAATTCTT)_2_ [31] sequences were performed on the basis of previous assignments by TOCSY and NOESY experiments. The assignments in the complexes were carried out by following the same procedure (Tables S2, S3 and S5). In particular, for G-quadruplexes, the guanine protons through the sequential NOE interactions and the inter-residue NOE connectivities between H1 and H8 resonances are characteristic of the three tetrads, thus confirming the conserved quadruplex structure. The assignment of the thymine H6 protons was followed by the NOE interactions with the methyl signals, which were easily recognized at high fields. The adenine aromatic protons were assigned through their ^1^H/^13^C coupling by HSQC experiments. Curaxin protons were assigned, in K-buffer solution pH 6.7, by an integrated series of 2D experiments, such as ROESY, TOCSY and COSY (Table S1). Peak assignment was carried out with Sparky programs.

### 3.3. Molecular Modeling Studies

The coordinates for the starting models of Pu22T14T23 and d(TTAGGGT)_4_ were obtained from the NMR structure deposited in the Protein Data Bank (accession code: 2L7V for Pu22T14T23 and 1NZM for d(TTAGGGT)_4_) [32,33].

The molecular docking calculations were performed by using the AutoDock 4.2 software [34]. The Lamarckian Genetic Algorithm [35], and the AutoDock Toolkit (ADT) [36] were used to further process the ligand and the Pu22T14T23 and d(TTAGGGT)_4_ models. The Gasteiger–Marsili charges [37] were added to the ligand by using ADT, and the phosphorus atoms in the DNA were parameterized by using the Cornell parameters. The solvation parameters were added to the system by means of the Addsol utility of AutoDock. The initial population consisted of 100 randomly placed individuals for each docking run, with a maximum number of 250 energy evaluations and an elitism value of 1, a mutation rate of 0.02 and a crossover rate of 0.80. The local search for the ligand was conducted by using 250 independent docking runs by applying the so-called pseudo-Solis and Wets algorithm with a maximum of 250 iterations per local search. The grid maps used in the actual docking process were calculated with Autogrid and cantered between the two K^+^ ions, with a grid dimensions of 80 × 80 × 80 Å (spacing of 0.01 Å). The docking results were scored by using an in-house version of the simpler intermolecular energy function based on the Weiner force field, and the results differing by less than 1.0 Å in positional root-mean-square deviation (rmsd) were clustered together and represented by the most favorable free energy of binding.

The best poses obtained from the previous phase were optimized through 5.0 ns of molecular dynamics (MD) using an OpenCL version of the GROMACS package with a modified version of the 53A6 GROMOS force field [38,39] and running on a dual-Xeon workstation (8 core) equipped with an NVIDIA GPU containing about 5000 CUDA^®^ cores. The systems were placed in the centre of a box with boundaries at 2.0 nm apart from all atoms. Then 3′-end and 5′-end terminal nucleotide topologies were modified according to Ricci et al. [40], the counterions (K^+^ ions) were random placed and SPC water molecules were added to the systems. The fully solvated systems were optimized through 100 ps of position restrained MD, followed by a heating ramp of short (100 ps) consecutive simulations at 50, 100, 150, 200, 250, and 300 K. The production simulations consisted of 5 ns of partially restrained MD at 310 K (time step of 0.002 ps).

The Lennard–Jones interactions were calculated by using a two-range switch interaction (cut-off radius of 0.9 and 1.1 nm), while the constraints were obtained by using the Lincs [41] and SETTLE [42] algorithms.

A Berendsen thermostat (coupling time of 0.1 ps) was applied to the systems [43], and the electrostatic interactions were calculated by using the Particle Mesh Ewald (PME) [44,45] method (Coulomb cut-off radius of 1.2 nm).

### 3.4. CD and Fluorescence

CD spectra were recorded on a Jasco J-810 spectropolarimeter equipped with a Peltier temperature control unit (Seelbach, Germany). The DNA solution (Pu22T14T23 or d(TTAGGGT)4 was transferred to a covered cell and ellipticity was recorded with a heating rate of approximately 0.4 °C·min^−1^. Simultaneously, CD spectra were recorded every 5 °C from 220 to 310 nm. The spectrum of the buffer was subtracted. Each sample was allowed to equilibrate at the initial temperature for 30 min before the melting experiment began. In all experiments, the concentration of DNA was kept constant (3 µM) whereas the concentration of the considered ligands was increased. The medium consisted of 25 mM KH_2_PO_4_ and 70 mM (Pu22T14T23) or 150 mM (d(TTAGGGT)_4_) KCl [46].

Molecular fluorescence spectra were measured with a JASCO FP-6200 spectrofluorimeter. The temperature was controlled at 20 °C using a water bath. The fluorescence spectra were monitored by using a quartz cuvette with a 10-mm path length, with the excitation and emission slits set at 10 nm, and the scan speed at 250 nm/min. Measurements were taken at 334 nm excitation wavelength. The buffer consisted of 25 mM phosphate buffer (pH 6.9) and 70 mM KCl. In all experiments, the concentration of curaxin was 3 µM, whereas the concentration of the considered DNA sequence was increased. The determination of the ratio ligand:DNA and the calculation of the binding constants were conducted from the fluorescence data recorded along titrations of ligands with DNAs by using the EQUISPEC program [47]. This program is based on the multivariate analysis of the whole spectra measured along the titration.

## 4. Conclusions

Curaxins are a class of substituted carbazoles that exert anticancer activity by complex and diverse mechanisms. Among curaxins, CBL0137 is under phase I clinical trials in patients with hematological malignancies and solid tumors.

Recent findings have claimed the involvement of DNA binding in curaxin antitumor activity [14,15].

In particular, numerous in vivo and in vitro experimental evidences, including inhibition of c-MYC expression, DNA methyltransferase inhibition, and chromatin remodelling, could be consistent with curaxin DNA G-quadruplex binding [4,14,15,17,18,19].

To gain a deeper insight into the potential DNA-binding properties of curaxin CBL0137 and to support the cellular evidences, we investigated its ability to interact with G-quadruplex DNA.

An NMR study was performed with the single repeat sequence of human telomers (TTAGGGT)_4_ and with Pu22T14T23, a model of the c-myc promoter Pu22 sequence. The results evidenced that the binding of curaxin to these G-quadruplex structures is significant. In the first case, curaxin was located over the G4 and G6 tetrads, with some exchange process between free and bound state at the level of G4. With the Pu22 sequence, the binding occurs at 3′-end and 5′-end, over the two external tetrads of G-quadruplex. The location at 3′-end appears more stable, while the interaction at 5′-end terminal is characterized by a higher mobility of the ligand. This behavior is in line with that observed for the telomere quadruplex (TTAGGGT)_4._ The results obtained from the detection of numerous NOE contacts between curaxin and both the G-quadruplex structures, were validated by molecular modeling studies.

The Kb values obtained for the 1:1 interaction of ligand **1** with Pu22T14T23 and d(TTAGGGT)_4_ are in the order of 10^6^ M^−1^, which indicating a significant interaction between this ligand and both structures. At high concentrations of curaxin, the formation of the 1:2 DNA:ligand complex is favored, especially in the case of Pu22T14T23. Curaxin is able to intercalate between the base pairs of B-DNA with a preference for CG-rich sequence. The relative small dimension of the flat aromatic portion of the molecule favors the entrance between the base pairs promoting the exchange among different sites. Overall, the results are in agreement with the literature’s available cellular data [4,14,15,17] and suggest that the interaction with G-quadruplex DNA may play a role in the anticancer activity of curaxin CBL0137. The molecular models here built for the complexes with DNA G-quadruplex structures could be a precious source of inspiration for the design of curaxin-related more active and selective DNA-binding ligands.

## Figures and Tables

**Table 1:** Intermolecular NOE and distances from modelling in the curaxin-d(TTAGGGT)_4_^a^ complexes.

	**ApG Binding Site**	
NOE		d (Å) ^b^
Curaxin	d(TTAGGGT)_4_	
1,8-H	A3H1′	4.60
2,7-H	A3H8	4.26
4,5-H	G4H1′	5.88
CH_3_ iso	A3H8	3.20
	**GpT Binding Site**	
1,8-H	G6H8	5.45
1,8-H	G6H1	4.36
1,8-H	T7Me	4.74
1,8-H	T7H1	3.13
4,5-H	G6H1	3.55
CH_3_ iso	G6H8	2.30
CH_3_ iso	G6H1	4.91
CH_3_ iso	T7H6	4.84
CH_3_ iso	G5H1′	5.91

^a^ Acquired at 25 °C in H_2_O-D_2_O (90:10 *v*/*v*), 25 mM K-phosphate buffer, 150 mM KCl and 1 mM EDTA, at pH 6.7. ^b^ Distances obtained by molecular modelling of the complex.

**Table 2:** Intermolecular NOEs and distances from modelling in the curaxin-Pu22T14T23 ^a^ complex.

**3′-Binding Site NOE**		**d (Å) ^b^**
Curaxin	Pu22T14T23	
4,5-H	G18H1	5.69
4,5-H	A24H2	6.60
CH_3_CO	G13H1	5.38
CH_3_CO	G22H1	4.68
CH_3 iso_	A24H2	6.21 ^c^
CH_3 iso_	A25H2	4.25
CH_3_CO	G18H1	2.26
**5′-Binding Site NOE**		
1,8-H	G7H1′	7.39 ^c^
2,7-H	G11H8	3.97
1,8-H	G11H8	5.34
CH_3 iso_	G20H8	2.75
CH_3 iso_	T4H1′	6.22 ^c^
CH_3_CO	G7H1	4.90
CH_3_CO	G11H1	3.45

^a^ Acquired at 25 °C in H_2_O-D_2_O (90:10 *v*/*v*), 25 mM KH_2_PO_4_, 70 mM KCl, pH 6.9. ^b^ Distances obtained by molecular modelling of the complex. ^c^ The long distance is explained with the mobility of the ligand and the tails of the nucleotide.

## Supplementary Materials

Supplementary Materials can be found at https://www.mdpi.com/article/10.3390/ijms22126476/s1.
