# Supplementary material for: Exploring the Interaction of Curaxin CBL0137 with G-Quadruplex DNA Oligomers
Source: Int J Mol Sci. 2021 Jun 17;22(12):6476. doi: 10.3390/ijms22126476 (PMC8234370; doi:10.3390/ijms22126476)
Supplement: Supplementary file 1 [file ijms-22-06476-s001.zip › ijms-1222936-supplementary.pdf]

International Journal of *Molecular Sciences*

MDPI

SUPPLEMENTARY MATERIALS

Exploring the Iiteraction of curaxin CBL0137 with G-quadruplex DNA oligomers

Sabrina Dallavalle [1,2], Luce M. Mattio [1], Roberto Artali [3], Loana Musso [1], Anna Aviñó [4], Carme Fàbrega[4], Ramon Eritja [4], Raimundo Gargallo [5] and Stefania Mazzini[1*]

1 Department of Food, Environmental and Nutritional Sciences (DEFENS), University of Milan (Università degli Studi di Milano), Milan, Italy; sabrina.dallavalle@unimi.it; luce.mattio@unimi.it; loana.musso@unimi.it; stefania.mazzini@unimi.it

2 National Institute of Fundamental Studies, Kandy 20000, Sri Lanka

3 Scientia Advice di Roberto Artali, 20832 Desio, MB, Italy; roberto.artali@scientia-advice.com

4 Institute for Advanced Chemistry of Catalonia (IQAC), CSIC, Networking Center on Bioengineering, Biomaterials and Nanomedicine (CIBER-BBN), Barcelona, Spain; aaagma@cid.csic.es; recgma@cid.csic.es

5 Department of Chemical Engineering and Analytical Chemistry, University of Barcelona, Barcelona, Spain; raimon_gargallo@ub.edu

* Correspondence: Stefania.mazzini@unimi.it

Contents:

Figure S1. Inter-residue NOE interactions between aromatic H8 and H1 imino protons of Pu22T14T23. Some intermolecular NOE are also reported.

Figure S2. Melting experiments of Pu22T14T23. (a) CD spectra measured along the melting experiment of Pu22T14T23. (b) CD spectra measured along the melting experiment of a mixture of Pu22T14T23 and curaxine (1:3 ratio). (c) Elipticity traces at 265 nm from both experiments.

Figure S3. Melting experiments of Pu22T14T23 in 5mM potassium phosphate buffer, pH 7.1, without KCl. In yellow CD spectra measured along the melting experiment of Pu22T14T23; in grey CD spectra measured along the melting experiment of a mixture of Pu22T14T23 and curaxine (1:3 ratio.

Figure S4. Titration of Pu22T14T23 with curaxin. (a) Experimental spectra measured along the titration. Numbers in inset indicate the DNA:curaxin ratio. (b) Experimental (symbols) and fitted (line) fluorescence at 450 nm. A 1:2 (DNA:curaxin) stoichiometry was used to fit the data.

Figure S5. Titration of $d(CGTACG)_2$ with curaxin. (a) Experimental spectra measured along the titration. Numbers in inset indicate the DNA:curaxin ratio. (b) Experimental (symbols) and fitted (line) fluorescence at 450 nm. A 1:1 (DNA:curaxin) stoichiometry was used to fit the data.

Figure S6. Titration of ss 5′-CTCTCTACTACCCTTCTGCTC-3′ with curaxin. Experimental spectra measured along the titration. Numbers in inset indicate the DNA:curaxin ratio.

Figure S7. (a) Schematic representation of $d(CGTACG)_2$; (b) imino protons region of the 1D NMR titration spectra $d(CGTACG)_2$ with curaxin.

Figure S8 .^{31}P spectra and schematic representation of of (a) $d(CGATCG)_2$ and (b) $d(AATT)_2$ duplexes at 15°C in 10 mM NaH_2PO_4, 100 mM NaCl, pH 7.0, 10% D_2O at different [drug]/[DNA] ratios.

Table S1. ^{1}H chemical shift assignments of curaxin in absence and in presence of $d(T_2AG_3T)_4$ and Pu22T14T23.

Table S2. ^{1}H chemical shift values of $d(T_2AG_3T)_4$ in the presence of curaxin.

Table S3. Selected ^{1}H chemical shift values for the complex of curaxin with Pu22T14T23.[a]

Table S4. Inter-residue NOE interactions of Pu22T14T23 in the complex with curaxin

Table S5. Selected ^{1}H chemical shift values for the complex of curaxin with and d(CGTACG)$_2$.

Table S6. Intermolecular NOE in the curaxin-d(CGTACG)$_2$ complex.

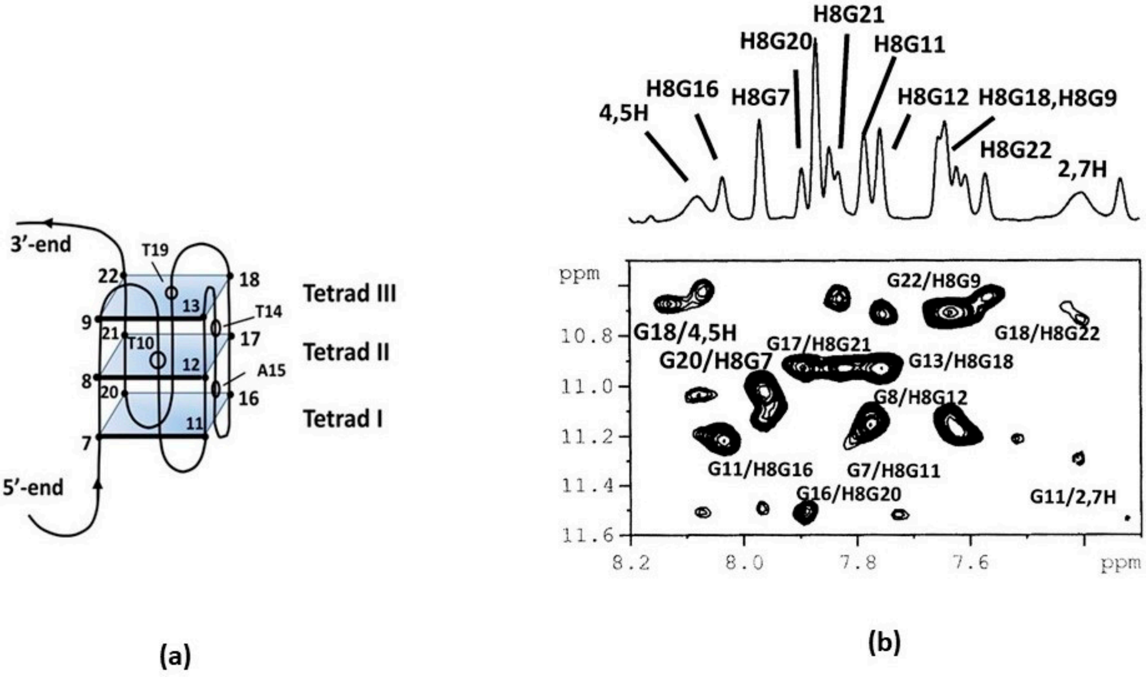

Figure S1. (a) Schematic representation of Pu22T14T23; (b) inter-residue NOE interactions between aromatic H8 and H1 imino protons of Pu22T14T23. Some intermolecular NOE are also reported.

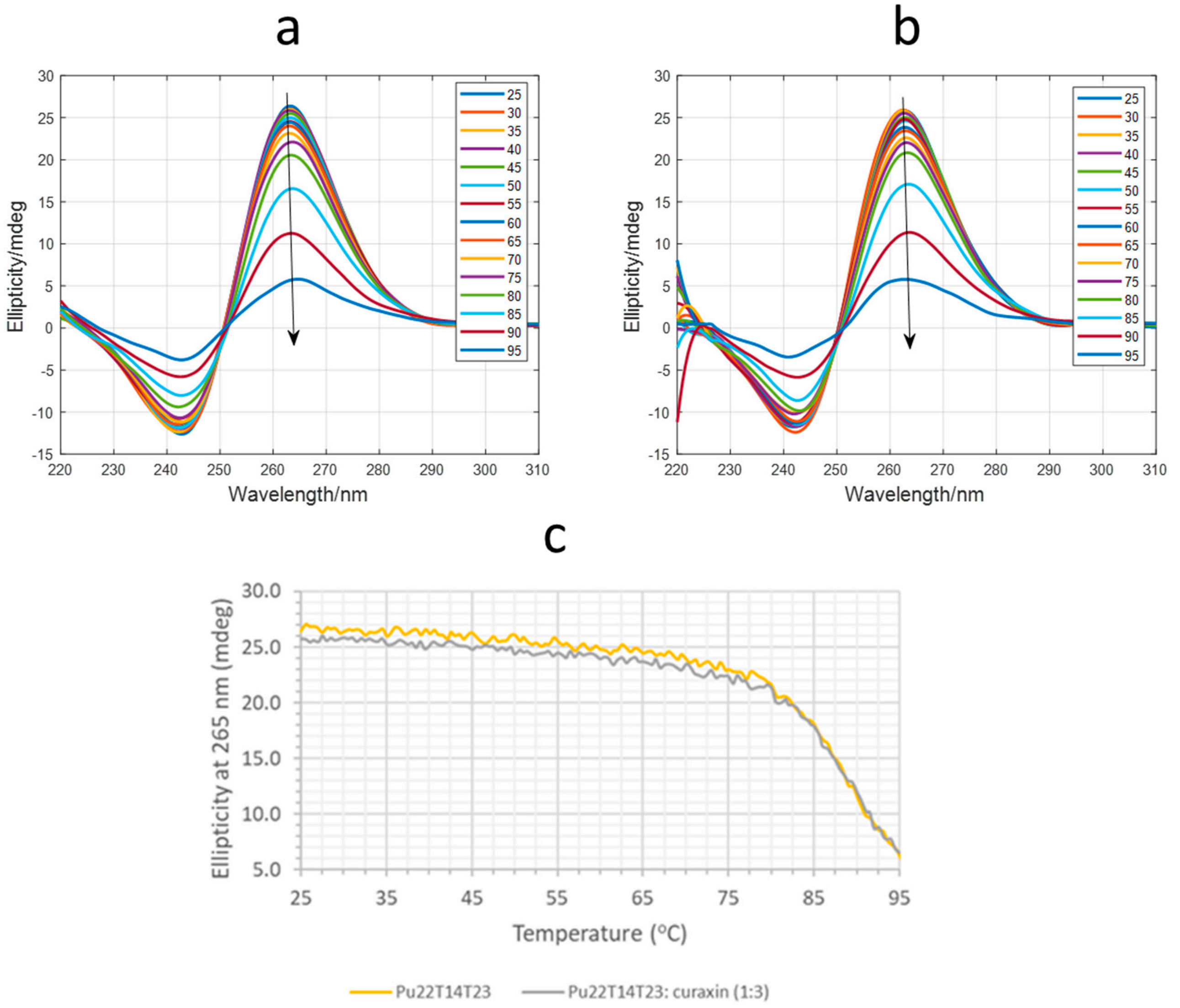

Figure S2. Melting experiments of Pu22T14T23. (a) CD spectra measured along the melting experiment of Pu22T14T23. (b) CD spectra measured along the melting experiment of a mixture of Pu22T14T23 and curaxine (1:3 ratio). (c) Elipticity traces at 265 nm from both experiments.

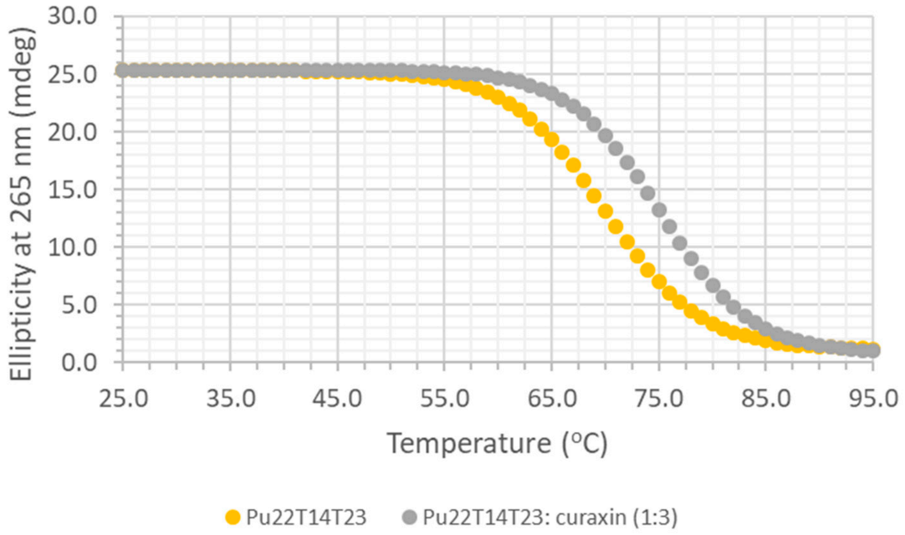

Figure S3. Melting experiments of Pu22T14T23 in 5mM potassium phosphate buffer, pH 7.1, without KCl. In yellow CD spectra measured along the melting experiment of Pu22T14T23; in grey CD spectra measured along the melting experiment of a mixture of Pu22T14T23 and curaxine (1:3 ratio)

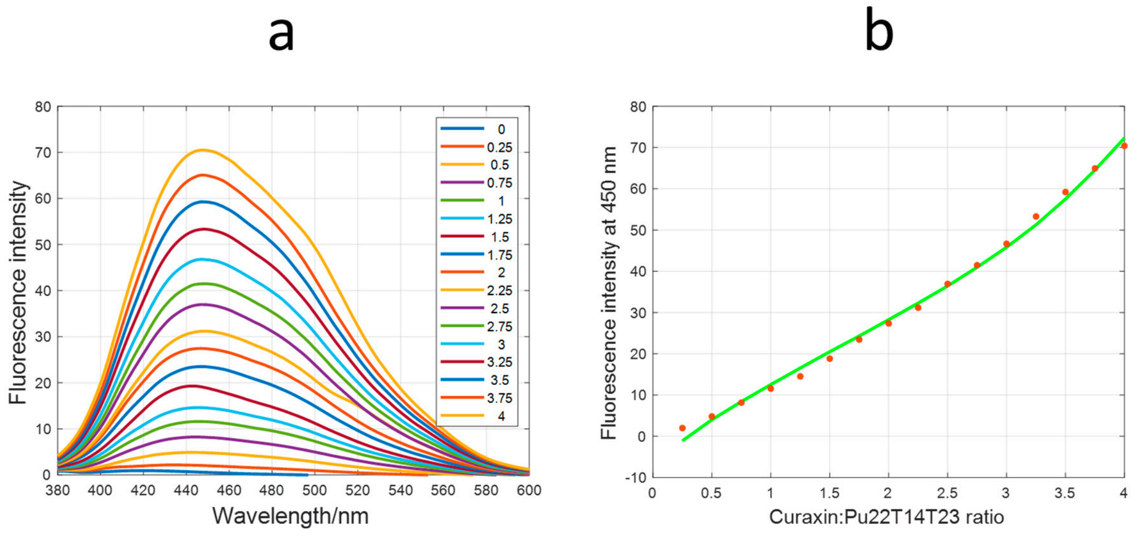

Figure S4. Titration of Pu22T14T23 with curaxin. (a) Experimental spectra measured along the titration. Numbers in inset indicate the DNA:curaxin ratio. (b) Experimental (symbols) and fitted (line) fluorescence at 450 nm. A 1:2 (DNA:curaxin) stoichiometry was used to fit the data.

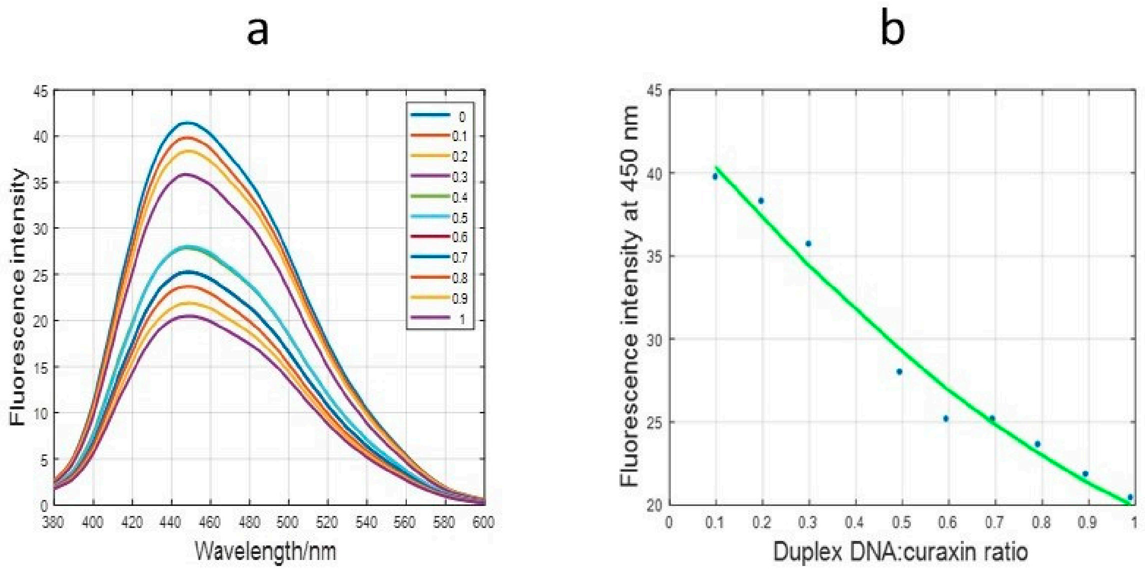

Figure S5. Titration of d(CGTACG)$_2$ with curaxin. (a) Experimental spectra measured along the titration. Numbers in inset indicate the DNA:curaxin ratio. (b) Experimental (symbols) and fitted (line) fluorescence at 450 nm. A 1:1 (DNA:curaxin) stoichiometry was used to fit the data.

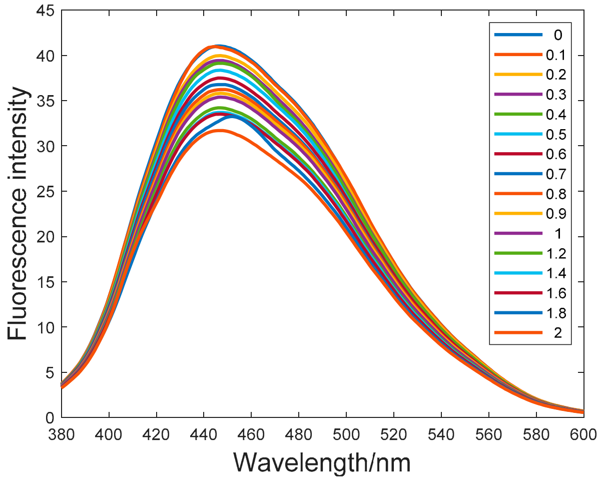

Figure S6. Titration of ss 5′-CTCTCTACTACCCTTCTGCTC-3′ with curaxin. Experimental spectra measured along the titration. Numbers in inset indicate the DNA:curaxin ratio.

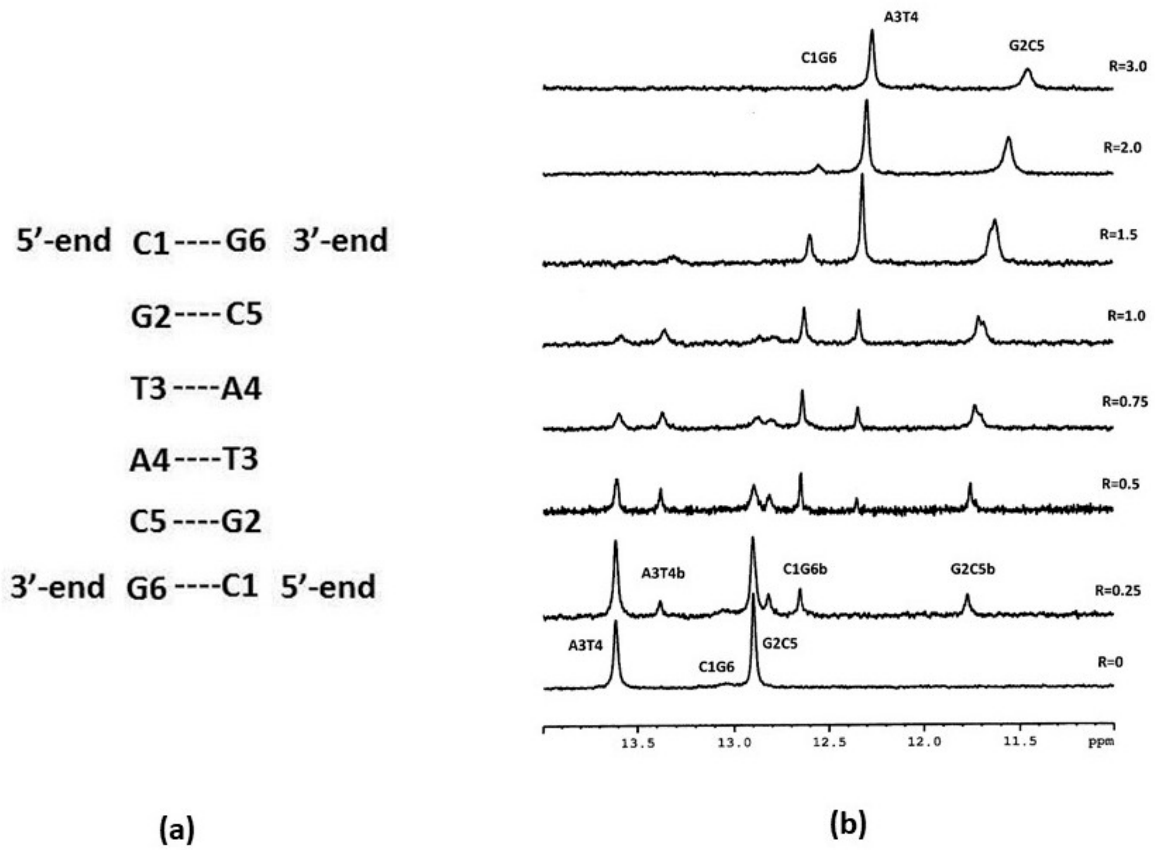

Figure S7. (a) Schematic representation of d(CGTACG)$_2$; (b) imino protons region of the 1D NMR titration spectra d(CGTACG)$_2$ with curaxin.

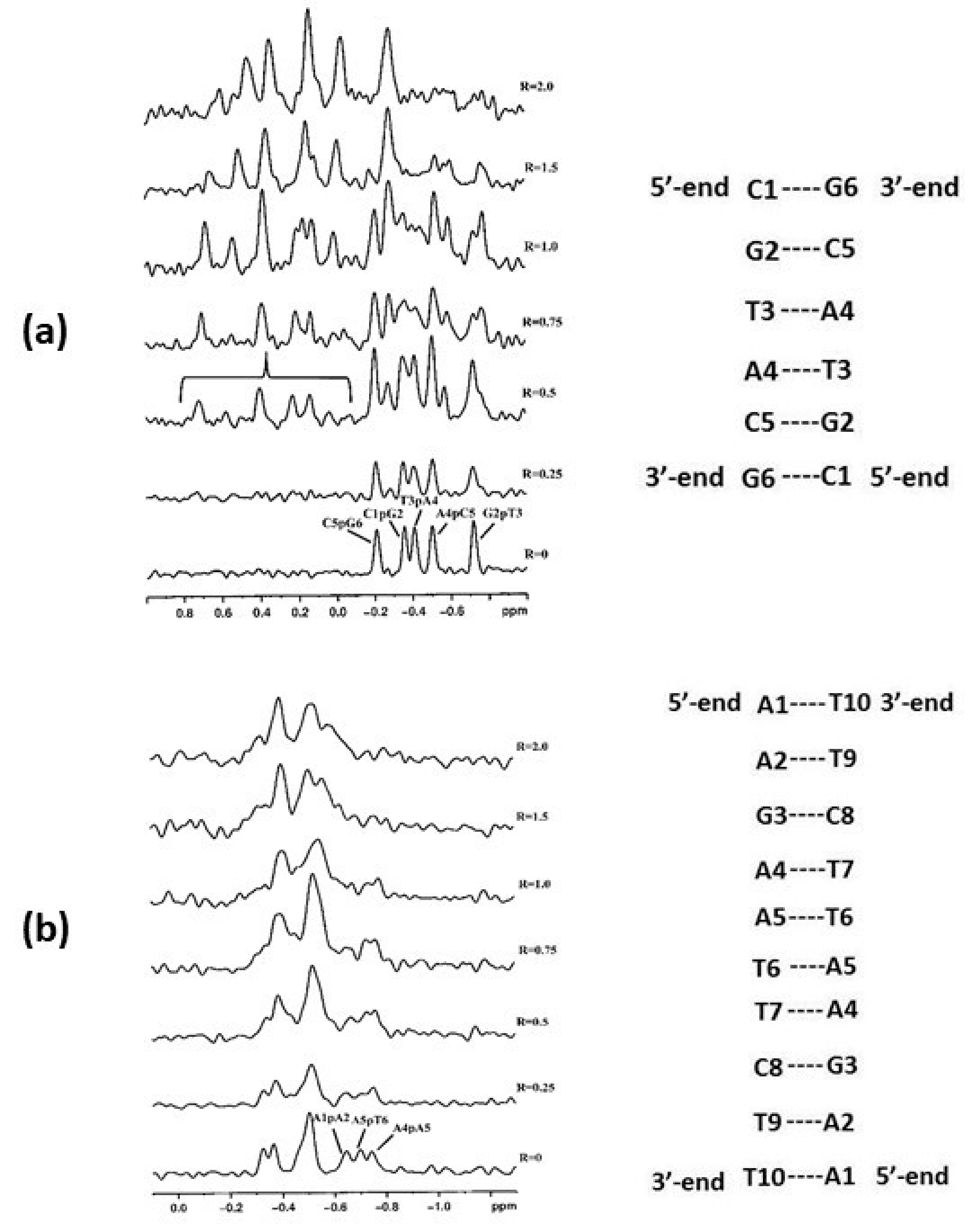

Figure S8 .^{31}P spectra and schematic representation of of (a) d(CGATCG)$_2$ and (b) d(AATT)$_2$ duplexes at 15°C in 10 mM NaH_2PO_4, 100 mM NaCl, pH 7.0, 10% D_2O at different [drug]/[DNA] ratios.

Table S1. [1]H chemical shift assignments of curaxin in absence and in presence of $d(T_2AG_3T)_4$ [a] and Pu22T14T23[b]

protons	δ curaxin free (ppm)	δ $d(T_2AG_3T)_4$ /curaxin (ppm)	Δδ[b]	δ Pu22T14T23 /curaxin (ppm)	Δδ[b]
1,8-H	7.50	7.00	-0.50	7.00	-0.50
2,7-H	8.05	7.44	-0.61	7.41	-0.64
4,5-H	8.41	8.17	-0.24	8.08	-0.33
$CH_2(9)$	4.55	4.19	-0.36	4.32	-0.23
CH_2	3.42	3.24	-0.18	3.32	-0.10
CH (isopropyl)	3.42	3.24	-0.18	3.32	-0.10
CH_3CO	2.68	2.26	-0.42	2.27	-0.41
CH_3 (isopropyl)	1.30	1.16	-0.14	1.19	-0.11

[a] Acquired at 25°C in H_2O-D_2O (90:10 v/v), 25 mM K-phosphate buffer, 150 mM KCl, 1mMEDTA, pH 6.7. [b] Acquired at 25°C in H_2O-D_2O (90:10 v/v), 25 mM KH_2PO_4, 70 mM KCl, pH 6.9. [c] $\Delta\delta = \delta_{bound} - \delta_{free}$

Table S2. ^1H chemical shift values of $d(T_2AG_3T)_4$ in the presence of curaxin[a]

$d(T_2AG_3T)_4$ /curaxin	H1 free	bound	Δδ	H8/H6 free	bound	Δδ	H2/Me free	bound	Δδ
T1	-	-	-	7.39	7.49	0.10	1.66	1.68	0.02
T2	-	-	-	7.30	7.31	0.01	1.76	1.77	0.01
A3	-	-		8.43	8.38	-0.05	8.09	n.d.	
G4	11.61	11.33	**-0.28**	7.95	7.78	**-0.17**	-	-	
G5	11.23	11.00	**-0.23**	7.79	7.61	**-0.18**	-	-	
G6	11.15	10.60	**-0.55**	7.70	7.69	-0.01	-	-	
T7	-	-	-	7.36	7.49	**0.13**	1.60	1.68	0.02

$d(T_2AG_3T)_4$ /curaxin	H1′ free	bound	Δδ	H2′/H2″ free	bound	Δδ
T1	6.00	6.11	0.11	2.10;2.34	2.27;2.27	0.17; -0.07
T2	6.23	5.93	-0.30	2.03;2.32	2.06;2.33	0.03;0.01
A3	6.28	6.26	-0.02	2.86;2.92	2.87;2.87	0.01;-0.05
G4	6.01	5.96	-0.05	2.67;2.91	2.60;2.82	-0.07;-0.03
G5	6.03	5.99	-0.04	2.66;2.74	2.60;2.82	-0.06;-0.09
G6	6.27	6.29	0.02	2.57;2.70	2.60;2.69	0.03;-0.01
T7	6.07	6.11	0.04	2.17;2.19	2.27;2.27	0.10;0.08

$d(T_2AG_3T)_4$ /curaxin	H3′ free	bound	Δδ	H4′ free	bound	Δδ
T1	4.64	n.d.	-	4.00	n.d.	-
T2	4.72	n.d.	-	4.06	n.d.	-
A3	5.10	5.08	-0.02	4.44	4.45	0.01
G4	5.05	4.98	-0.07	4.49	4.45	-0.04
G5	5.04	4.98	-0.06	4.51	4.45	-0.06

G6	4.91	4.98	0.07	4.52	4.46	-0.06
T7	4.49	5.02	0.03	4.23	4.21	-0.02

d($T_2AG_3T)_4$ /curaxin	H5′/H5″ free	bound	Δδ
T1	3.65;3.65	n.d.	-
T2	3.90;3.90	n.d.	-
A3	4.15;4.10	4.12;4.12	-0.03, 0.02
G4	4.27;4.27	4.24;4.24	-0.03; -0.03
G5	4.30;4.30	4.27;4.27	-0.03; -0.03
G6	4.27;4.27	4.24;4.24	-0.03; -0.03
T7	4.07;4.07	4.12;4.12	0.05; 0.05

[a] Acquired at 25°C in 25 mM KH_2PO4, 150 mM KCl and 1 mM EDTA, pH 6.7, 10% D_2O, R=2.0

Table S3 . Selected ^{1}H chemical shift values for the complex of curaxin with Pu22T14T23.[a]

	H1/H2/Me	Δδ[b]	H6/H8	Δδ
T4	1.79	+0.14	7.33	+0.12
G5	n.d.	-	8.24	+0.24
A6	7.97	+0.17	n.d.	-
G7	11.20	**- 0.56**	7.97	-0.07
G8	10.94	- 0.28	7.60	-0.12
G9	10.30	-0.30	7.71	-0.11
T10	n.d.	-	n.d.	
G11	11.23	**-0.48**	7.78	-0.21
G12	11.17	-0.33	7.76	-0.24
G13	10.70	**-0.36**	7.78	-0.08
T14	1.94	+0.02	7.64	-0.01
A15	8.36	-0.02	8.55	+0.02
G16	11.50	**- 0.40**	8.04	-0.07
G17	10.93	- 0.32	7.63	- 0.17
G18	10.64	**- 0.38**	7.66	- 0.13

T19	2.00	+0.01	7.86	0.00
G20	11.03	-0.24	7.90	0.00
G21	11.13	-0.24	7.83	-0.08
G22	10.72	**-0.32**	7.57	-0.04
T23	1.30	-0.18	7.09	-0.05
A24	7.23	+0.13	n.d.	-
A25	7.60	+0.21	n.d.	-

[a] Measured at 25°C in ppm (δ) from external DSS. Solvent H_2O-D_2O (90:10 v/v), 25 mM K-phosphate buffer, 70 mM KCl, pH 6.9, *R* = 2.0.

[b] $\Delta\delta = \delta_{bound} - \delta_{free}$

Table S4. Inter-residue NOE interactions of Pu22T14T23 in the complex with curaxin[a]

G-tetrad I	*G-tetrad II*	*Tetrad III*
G7H1-G11H8	G8H1-G12H8	G9H1-G13H8
G11H1-G16H8	G12H1-G17H8	G13H1-G18H8
G16H1-G20H8	G17H1-G21H8	G18H1-G22H8
G20H1-G7H8	G21H1-G8H8	G22H1-G9H8

[a] Acquired at 25°C in H_2O-D_2O (90:10 v/v), 25 mM K-phosphate buffer, 70 mM KCl, pH 6.9; R=2.0

Table S5. Selected ^{1}H chemical shift values for the complex of curaxin with and d(CGTACG)2[a]

**"CG"**	H2/H5/CH_3	Δδ[b]	H6/H8	Δδ[b]
C1	5.44		7.35	-0.12
G2	-	-	7.53	-0.12
T3	1.61		7.15	-0.07

A4	6.97		8.06	-0.06
C5	5.52		7.32	-0.11
G6	-		7.79	-0.09
	NH			
C1G6	12.45	-0.60		
G2C5	11.45	**-1.45**		
T3A4	12.27	**-1.34**		

[a] Measured at 15°C in ppm (δ) from external DSS. Solvent H_2O-D_2O (90:10 v/v), of 0.1 M NaCl and 10 mM sodium phosphate buffer solution, pH = 7.0; R = 3.0. [b]$\Delta\delta = \delta_{bound} - \delta_{free}$; [c] Very broad signal.

Table S6. Intermolecular NOE in the curaxin-d(CGTACG)$_2$[a] complex

**NOE**	
**Curaxin**	d(CGTACG)$_2$
1,8-H	G2H8
2,7-H	G2H8
4,5-H	G2H8
CH_3 iso	A4H8
CH_3CO	A4H8
1,8-H	A4H8
2,7-H	A4H8
1,8-H	A4H8
2,7-H	T3H6
4,5-H	T3H6

[a] Measured at 15°C in ppm (δ) from external DSS. Solvent H_2O-D_2O (90:10 v/v), of 0.1 M NaCl and 10 mM sodium phosphate buffer solution, pH = 7.0; R = 3.0.
